# Faking It Isn’t Making It: Research Needs Spontaneous and Naturalistic Facial Expressions

**DOI:** 10.1007/s42761-025-00320-1

**Published:** 2025-07-24

**Authors:** Amy Dawel, Eva G. Krumhuber, Romina Palermo

**Affiliations:** 1 School of Medicine and Psychology, The Australian National University, Canberra, ACT 2600 Australia; 2 Department of Experimental Psychology, University College London, London, UK; 3 School of Psychological Science, The University of Western Australia, Perth, Australia

**Keywords:** Facial expression, Emotion perception, Naturalistic, Genuine, Posed, Artificial

## Abstract

Facial expressions play a pivotal role in shaping social interactions. However, the conceptualization of facial expressions as direct readouts of internal emotional experience has led to the conflation of three distinct question types. Specifically, there is confusion between questions concerning: (Q1) the production of facial expressions, (Q2) how accurately perceivers interpret expressors’ internal emotions from their outward expressions, and (Q3) perceiver responses to the outward appearance of expressions independent of the expressor’s internal emotional state. The disentanglement of these three question types highlights that, because the facial stimuli traditionally used in research are posed rather than reflective of internal emotions, they can only test perceiver responses (Q3), though they have often been interpreted as measures of perceptual accuracy (Q2). Moreover, due to their exaggerated and prototypical nature, these stimuli fail to capture the nuance and complexity of real-world expressions, potentially leading to ecologically invalid findings even for Q3. New data presented here also suggest that many of these stimuli are *not* perceived as genuinely emotional and may appear unnatural. We review evidence demonstrating that stimuli that are naturally- or spontaneously-elicited and/or appear genuinely emotional can produce different findings than traditional posed stimuli. Fortunately, naturalistic and spontaneous expression stimuli are now readily available for the field to move forward. We conclude with seven recommendations for advancing facial expression research.

People generally feel that they can directly “read” others’ emotions from their facial expressions. This intuitive sense aligns with Paul Ekman’s basic emotions theory (BET; Ekman, 1992; Ekman & Cordaro, 2011). BET asserts that, for a small number of basic emotions, facial expressions are direct readouts of true internal emotion, implying that one’s internal emotions are expressed through the face without any voluntary control or moderation. However, there is evidence this is not the case. Display rules, a concept introduced by Ekman et al. (1969), explains how norms guide the regulation of emotional expressions in different social and cultural contexts (Dawel et al., 2023; Matsumoto et al., 2008; Monaghan et al., 2025). Supporting the enaction of display rules, the human face is innervated by two systems: one involuntary, activated by genuine feelings of emotion, and another voluntarily controlled, used for posing emotions for social and communicative reasons (Rinn, 1984; also see Matsumoto & Wilson, n.d., for an introduction to facial anatomy). Direct evidence for the dual-innervation of human expressions comes from studies demonstrating a double-dissociation in patients with neurological damage. In patients with emotional paresis, a smile can be posed intentionally but will not be elicited spontaneously by a pleasant event, whereas the reverse is true in patients with volitional paresis (Hopf et al., 1992; Sim et al., 2005; Urban et al., 1998). For most of us though, facial expressions involve the integrated action of these two systems, meaning facial behavior is often, if not always, regulated by voluntary control, with “pure” readouts of emotion being rare. This perspective aligns more closely with alternative theorizing, which views facial displays as having a more holistic communicative function (Barrett, 2017; Crivelli & Fridlund, 2018; Jack & Schyns, 2015).

Despite this, BET’s significant influence and pervasive claim that facial expressions are direct readouts of internal emotion has led to confusion among three types of questions: (Q1) those about expression production, (Q2) those testing perceptual accuracy—the match between the perceiver’s interpretation and the expressor’s emotional state, and (Q3) those focused solely on the perceiver’s interpretation and other responses to the physical appearance of the face, regardless of the expressor’s internal emotional state. Part of the confusion stems from the predominant use of posed facial expressions. Since these posed stimuli do not reflect internal emotions, we argue research based on these stimuli can only address perception-driven responses (Q3), though they are often misinterpreted as testing perceptual accuracy (Q2). Moreover, the field’s heavy reliance on certain types of stimuli compromises the ecological validity of past findings concerning perception-driven responses (Q3). Specifically, since the dominant stimuli are artificially posed and do not appear genuinely emotional—a claim supported by new data presented here—findings based on these stimuli may not generalize to real-world social behavior. A brief review of studies comparing responses to naturalistic and spontaneous expressions with those to posed ones suggests that the over-reliance on posed expressions has likely distorted scientific understanding, necessitating conceptual replication of established findings using ecologically-valid stimuli. Finally, we discuss practical steps scientists can take to address these issues, including developing and using more naturalistic and spontaneous expression stimuli, elicited by internal emotions. We conclude with seven recommendations for advancing the field.

## Disentangling the Three Types of Questions about Facial Expressions

**Fig. 1:**
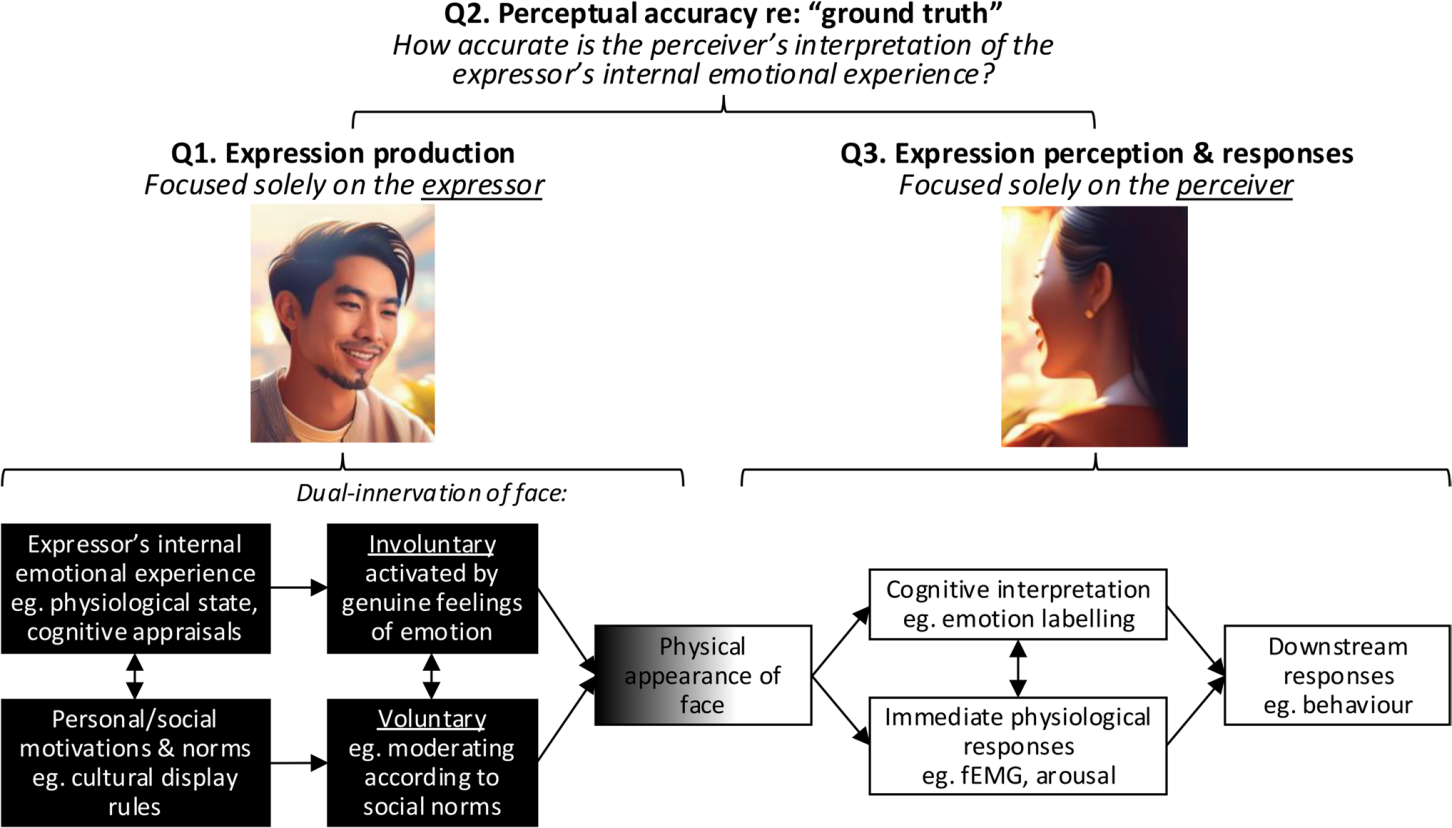
Disentangling the Three Types of Questions about Facial Expressions. *Note*. Faces generated using Adobe Firefly

Figure 1 outlines the three types of questions and their connection to the dual-innervation model of expression production and perceiver responses. In this section, we briefly define the question types and their key implications for the literature. To concretize these distinctions, Table 1 offers example studies that address each question type.

**Table 1:** Examples of Studies Addressing Questions about (Q1) Facial Expression Production, (Q2) Perceptual Accuracy, and (Q3) Perception and Responses (with Example Studies)

	Q1. Facial expression production	Q2. Perceptual accuracy	Q3. Perception and responses
Fundamental questions	How predictably do facial expressions correspond to internal emotional states? (for meta-analysis and debate see: Durán & Fernández-Dols, 2021, 2023; Witkower et al., 2023)	How accurately can perceivers decode expressors’ internal emotional state from their facial display? (McLellan et al., 2010; Miles & Johnston, 2007)	How do physical cues influence the perception of emotion in facial expressions? (e.g., tears, Küster et al., 2023; the Duchenne marker, Miller et al., 2022; and blushing, Thorstenson et al., 2020)
Context effects	How do facial expressions vary across social settings? (e.g., smiling in social vs non-social situations; Fernández-Dols & Ruiz-Belda, 1995; Fridlund, 1991; Matsumoto & Willingham, 2006)	How does other visual information contribute to perceptual accuracy? (e.g., bodily expressions, Aviezer et al., 2012)	How do contextual cues, such as body posture and visual scenery, influence the perception of emotion in faces? (for reviews see: Aviezer et al., 2017; Steward et al., 2024)
Cross-cultural/country differences	Are there cultural differences in the facial expressions people produce? (e.g., automated coding of expression morphology across 31 countries, Srinivasan & Martinez, 2018)	Is perceptual accuracy reduced for other cultures compared to one’s own? (see results for spontaneous expressions in Elfenbein & Ambady, 2003)	How does culture influence the interpretation of facial expressions? (for review and debate of cross-cultural emotion labelling see: Ekman, 1994; Russell, 1994; for cross-cultural mental representations see: Jack et al., 2012)
Individual differences	How do clinical conditions affect facial expression production? (e.g., in autism spectrum disorder, Trevisan et al., 2018; and visual blindness, Valente et al., 2018)	How is perceptual accuracy impacted in clinical and neuropsychological conditions? (e.g., in schizophrenia, Davis & Gibson, 2000; depression, Douglas et al., 2012; and traumatic brain injury, McLellan & McKinlay, 2013)	How does expression perception vary with individual differences in other abilities (e.g., vocal emotion labelling and empathy, Palermo et al., 2013, 2018) and in clinical conditions? (e.g., in psychopathy, Dawel et al., 2012; and schizophrenia, Mewton et al., 2025)

### Q1. Expression Production

Questions about expression production centre exclusively on the expressor. The main focus has been *how* the expressor’s internal emotion is reflected in the physical appearance of their face and whether this relationship is consistent, making facial expressions reliable indicators of internal emotion. While most researchers agree that human emotional experience includes physiological components, such as autonomic nervous system changes, and cognitive components, such as situational appraisals and self-reportable feelings, there is debate about how these internal experiences relate to outward expression (Adolphs et al., 2019; Barrett et al., 2019). Accordingly, research addressing this question type has examined relationships such as: if someone shows physiological markers associated with positive affect (e.g., right-sided neural activation; Davidson et al., 1990) or reports feeling happy (Hall & Horgan, 2003), are they more likely to smile than not, or display a Duchenne^1^ smile?

Importantly, as predicted by the dual-innervation model, while there is general agreement that certain facial displays are more likely to accompany specific emotions than expected by chance (e.g., smiling is more likely to co-occur with happiness than without; Krumhuber & Kappas, 2022), this relationship is weak and variable (Barrett et al., 2019; Witkower et al., 2023; cf. Durán & Fernández-Dols, 2021, 2023). Emotions can be felt internally without being outwardly expressed (Butler et al., 2003) and people often display facial expressions in the absence of the corresponding internal emotion or to mask an alternative emotion (Ansfield, 2007; Krumhuber & Manstead, 2009; Zloteanu & Krumhuber, 2021), such as smiling to hide anger at one’s boss. The expression of emotions is also influenced by the presence of others and one’s relationship with them (Fernández-Dols & Ruiz-Belda, 1995; Fischer et al., 2003; Lloyd & Summers, n.d.; Ruiz-Belda et al., 2003), illustrating how display rules may guide regulation across different social contexts. Expressive displays may also vary across individuals (Cohn et al., 2002) and cultures (Talhelm et al., 2019). Thus, studies on production indicate there is not a 1:1 correspondence between internally experienced and outwardly expressed emotion. That is, facial expressions are often *not* direct readouts of internal emotion.

### Q2. Perceptual Accuracy

Questions about perceptual accuracy examine the alignment between the expressor’s internal emotion and the perceiver’s interpretation of the expressor’s facial expression. These questions are challenging to tackle because they require stimuli that are accompanied by information about expressors’ emotional experiences, which is often missing from databases (Krumhuber et al., 2016). Additionally, some researchers argue that it may not be possible to truly know how expressors are feeling, as self-reports are often biased and may lack insight (Barrett et al., 2019; Luminet et al., 2021). One alternative is to use the eliciting context (e.g., film clips designed to elicit specific emotions like happy, sad, or disgust) as a proxy for measuring expressors’ internal emotional states, but this assumes that all people have the same emotional reaction to a given situation, which is untrue (Kuppens & Tong, 2010). Despite these critiques, pursuing this challenging line of research can provide valuable insights. For instance, individual differences could inform real-world applications. People who excel in this area may be well-suited for roles such as psychologists or other human-to-human work, while those with impairments may benefit from interventions that enhance their social engagement (e.g., in schizophrenia spectrum disorder; Mewton et al., 2025).

### Q3. Expression Perception and Responding

This type of question focuses exclusively on the perceiver’s interpretation of and responses to the physical appearance of a face, without considering how the expressor truly feels (i.e., ignoring the expressor’s internal experience of emotion, which may or may not align with their outward facial expression, as per Q1). Perceiver-focused questions are important because people respond to what they observe, *not* what someone else is feeling internally. These studies provide insight into how various physical cues in the face drive perceiver interpretations of emotion and other responses. While the traditional focus has been on facial morphology (Matsumoto & Wilson, n.d.), it is now understood that gaze and head direction, coloring (e.g., blushing), and tears are also important cues to emotion (Kret, 2015; Küster et al., 2021). The use of physical cues to judge emotion can also differ across individual perceivers and cultural groups (Elfenbein & Ambady, 2003; Thibault et al., 2012; Uono & Hietanen, 2015).

An enormous number of studies have tested expression perception and responding (Barrett et al., 2019; Dawel et al., 2022), examining a wide variety of outcomes, including emotion labelling, physiological responses, and downstream behavioral responses like willingness to engage with or help others (see Zhang & Winkielman, n.d., for a review of response types).

Many of these studies aimed to measure expression recognition ability (e.g., percentage correct on a forced-choice labelling task) and have often been interpreted as assessing perceptual accuracy (Q2). However, most studies purportedly about ‘recognition’ did not actually test participants’ ability to recognize expressors’ internal emotions, as the target expressions were artificially posed^2^ (see ‘*Issues Concerning Traditional Expression Stimuli*’ section for further elaboration). True perceptual accuracy tasks test the alignment between the perceiver’s response and the expressor’s internal emotion; for this purpose, it is insufficient to simply test responses to the physical appearance of a face.

To advance the field, it is crucial to distinguish perception studies (Q3) from those addressing perceptual accuracy (Q2). Isolating evidence that pertains only to perception (rather than accuracy) shifts the focus of outcome measures from Q3 toward agreement or consensus. This shift provides a foundation for exploring perceptual consensus as a distinct and valuable subject of inquiry. For instance, reframing traditional ‘recognition accuracy’ studies as tests of consensus opens new questions about the moderating factors that drive divergence from consensus (e.g., cross-cultural and individual differences) without requiring a ground truth label. Furthermore, given the pivotal role of facial displays in guiding human interactions (Adams et al., 2017), shared interpretations of these displays may contribute to effective social coordination (Baek & Parkinson, 2022). Conversely, low consensus between perceiver’s could lead to misunderstandings and inconsistent responses.

However, the field faces another major challenge in reinterpreting studies testing Q3. Specifically, as we elaborate in the next section of this article, *‘Issues Concerning Traditional Expression Stimuli’,* much of the perception/response evidence is derived from limited stimulus sets featuring highly standardized posed expressions. In addition to the lack of internal emotion that makes these stimuli unsuitable for testing Q2, these stimuli may also be unsuitable for testing Q3 because they do not look like the types of expressions that people display in the real-world. Thus, perceiver consensus and responding to them may not reflect everyday social behavior.

### An Additional Challenge to Disentangling the Questions

A further challenge to effectively distinguishing studies of Q3 from those testing Q2 is that people—including researchers—may intuitively feel they can read others’ emotions from their facial expressions. As a result, there is a challenge in arguing that perception can (and should) be separated from the expressor’s experience. However, this seems worthwhile. For instance, related literature on facial first impressions distinguishes between questions about how people perceive and respond to the physical features of faces (Q3) (e.g., What makes a face appear trustworthy? How does looking trustworthy influence perceiver behavior? Olivola et al., 2014; Sutherland et al., 2013) and the accuracy of those perceptions (Q2) (e.g., Do people who look trustworthy behave in more trustworthy ways? Jaeger et al., 2022). This line of work has revealed perceptual accuracy for trustworthiness is low (Foo et al., 2022), but nevertheless perceptions of trustworthiness influence perceiver responses in important ways, including electoral voting and criminal sentencing decisions (Olivola et al., 2014). Thus, the case of trustworthiness highlights the benefits of studying perception (Q3) separately from perceptual accuracy (Q2).

## Issues Concerning Traditional Expression Stimuli

This section shifts focus to five key issues with facial expression stimuli that have dominated this field, explaining why they can only address Q3 (not Q1 or Q2) and, moreover, why they may not be well-suited even for this purpose. In summary, the main issues are that the most commonly used stimuli: (1) come from a small number of sources; (2) primarily feature posed expressions, which are often perceived as non-genuine and unnatural (see later section ‘*Assessing the Perceived Genuineness and Naturalness of Traditional Stimuli*’); (3) are highly-standardized and lack important emotional cues such as tears, facial coloring (e.g., blushing), and variation in eye-gaze and head direction; (4) were developed primarily in Western contexts and lack demographic diversity; and (5) have been validated primarily through high perceiver agreement on emotion labelling tasks, which is an insufficient criterion. Ultimately, these stimuli can only address perception (Q3) because they were not elicited by an internal emotional experience. They are unsuitable for testing production (Q1) or perceptual accuracy (Q2) as there is no internal emotion to compare with the expressor’s outward display (Q1) or the perceiver’s response (Q2).

### Limited Sources

The first issue is that the literature has relied too heavily on a small number of stimulus sets. Our recent systematic survey reveals that just four stimulus sources were used across two-thirds of facial perception studies in psychology and neuroscience from 2000 to 2020 (Dawel et al., 2022). Namely, Ekman and colleagues’ Pictures of Facial Affect (PoFA; (Ekman & Friesen, 1976) and Japanese and Caucasian Facial Expressions of Emotion (JACFEE; Matsumoto & Ekman, 1988), the Radboud Faces Database (RaFD; Langner et al., 2010), the Karolinska Directed Emotional Faces (KDEF; Lundqvist et al., 1998), and the NimStim database (Tottenham et al., 2009). These stimulus sets—which were developed in line with best practice at the time and generously shared—have played a key role in moving the field forward, with extensive use across diverse fields such as social, cognitive, clinical, and developmental psychology, psychiatry, neuroscience, economics, and computer science (Dawel et al., 2022). However, while offering rigorous standardization and high perceiver consensus on the emotion categories signalled, as the next sections elucidate, these stimuli may have limited scientific advancement in important ways, ultimately undermining the ecological validity of findings.

### Artificially Posed

The second issue, which is a particular threat to ecological validity, is that these four stimulus sources all show mostly posed rather than spontaneous or naturalistic expressions. Ekman and colleagues’ stimuli (Ekman & Friesen, 1976; Matsumoto & Ekman, 1988) and the RaFD (Langner et al., 2010) used the Directed Facial Action Task (Ekman, 2007) which, without mentioning an emotion label, instructs posers to configure their face in specific ways that had been derived from studying the morphology of real-life expressions (e.g., for disgust: “Wrinkle your nose, let your lips part. Pull your lower lip down. Let your tongue move forward in your mouth but you don’t need to stick it out.” Ekman, 2007, p. 51). The KDEF (Lundqvist et al., 1998) and NimStim (Tottenham et al., 2009) databases were less directly specified, but nevertheless created by instructions to pose a named emotion. The only notable exception to the posing of stimuli within these sets is that the PoFA happy expressions were captured during spontaneous interactions with the experimenter/photographer (Ekman, 1980). As a result of these posing methods, these databases typically comprise exaggerated and homogenous expressions, which do not reflect the variety of “in-the-wild” real-life expressions (Barrett et al., 2019).

### Highly Standardized

A related issue is that the standardized way in which these stimuli were produced also prevented them from including the nuanced and complex characteristics of everyday expressive displays. Indeed, spontaneous and naturalistic facial actions have different morphological characteristics than posed ones (Cohn & Schmidt, 2004; Küster et al., 2021). Posed stimuli also omit non-morphological cues to emotion such as facial coloring and tears. While two of the four databases present some variation in eye-gaze and head direction (the KDEF varies head direction and the RaFD varies both), these are still standardized to show only certain viewpoints (e.g., frontal, three-quarter and profile, with no change in head pitch or roll) and may omit naturally-occurring combinations. For instance, none of these databases show sad expressions with downward gaze and/or head direction, though both cues are perceived as signalling sadness (Haile et al., n.d.; Semyonov et al., 2021). Missing these cues is important, as they can influence perceiver responses (Kret, 2015).

Together, the issues of posing and standardization present a strong counterargument to one justification for using traditional stimuli. This justification asserts that, despite being posed, these stimuli replicate the physical properties of emotion-elicited expressions, achieving ecological validity (Matsumoto & Wilson, n.d.). This claim specifically pertains to Ekman and colleagues’ sets (Ekman & Friesen, 1976; Matsumoto & Ekman, 1988), which rigorously identified the morphological features for signalling basic emotions—anger, disgust, fear, happiness, sadness, and surprise—and translated them into expression stimuli using the Directed Facial Action Task (Ekman, 2007). However, this task generates strong and prototypical expressions that omit non-morphological cues and lack the complexity and nuance of real-life displays. As we will see in the section ‘*Assessing the Perceived Genuineness and Naturalness of Traditional Stimuli*’, many of these expressions are also perceived as faking emotion and unnatural.

### Lack of Diversity

The expression stimulus sets most widely used in psychology and neuroscience were developed primarily in Western contexts and predominantly feature White young adult faces (Dawel et al., 2022). Similarly, a recent review of affective computing stimulus sets highlights a persistent lack of diversity in this field (Verhoef & Fosch-Villaronga, 2023). This underrepresentation of diversity is problematic because emotion expression and perception vary significantly across cultures (Elfenbein & Ambady, 2003; Jack et al., 2012; Srinivasan & Martinez, 2018), and differences in facial structure and skin texture across cultural, gender, and age groups can influence emotion recognition (Hedgecoth et al., 2023). The limited diversity in facial stimuli has reinforced the WEIRD (White, Educated, Industrialized, Rich, and Democratic) bias in psychological research (Henrich et al., 2010), limiting the generalizability of findings.

### Validation through Consensus Labelling

A final point of note is that, in addition to any morphological criteria, validation of these databases has often relied on high perceiver agreement about the emotions displayed. However, while useful, consensus alone is an inadequate criterion. Even highly unrealistic stimuli like emoticons can generate high consensus in an emotion labelling task, illustrating the insufficiency of this criterion.

## Assessing the Perceived Genuineness and Naturalness of Traditional Stimuli

As noted previously, a key argument for using lab-developed expression stimuli to test perception and responses (Q3) is that they could, in theory, replicate the physical features of emotion-elicited expressions, thereby achieving ecological validity. While the ideal test of this argument is whether stimuli perfectly match the physical features of naturally occurring expressions, when this claim is made (as in Matsumoto & Wilson, n.d.), we might hope that they are perceived as genuinely signalling internal emotions. However, visual observation suggests that the exaggerated and prototypical nature of traditional stimuli may make them appear caricatured and unnatural to participants. This is an important empirical question, yet there is little data on whether the most commonly used stimuli are perceived to be genuinely emotional and natural.

To address this gap, we asked participants to rate how genuinely emotional and natural they perceived the most used expression stimuli to be. We used full or representative samples of stimuli from each of the four most used databases (PoFA, RaFD Caucasian adults, KDEF, NimStim) and tested the six basic emotions included in all four databases (anger, disgust, fear, happiness, sadness, surprise). For both rating tasks, each stimulus was rated by 16 perceivers to ensure stimulus-level ratings were reliable (DeBruine & Jones, 2022). The full rating method, data, and analysis code are available at: https://osf.io/gtnx5/?view_only=0451d7def4864b558ad261db56fd11ab.

To assess perceptions of emotional genuineness, we used the rating task developed by Dawel et al. (2017), which defines genuine and faked/acted/posed emotional expressions as follows:

*“Sometimes people’s faces express emotions they genuinely feel. Other times facial expressions are faked, acted or posed (e.g., to be polite, or for other social reasons). An example of a genuine expression is when somebody smiles and they really feel happy, like when they get a present or hear something funny. An example of a faked expression is when somebody smiles for a school photo, without feeling any emotion. Or a parent playing a game with their child may put on a ‘scared’ face to pretend fear, without actually feeling afraid. People often also pose expressions as part of everyday conversation, or to communicate empathy or understanding. Some expressions are also posed to mask other emotions (e.g., putting on a “happy” smile to mask disappointment).”*

Perceivers rated genuineness on a scale from −7 (completely fake) to +7 (completely genuine), with a neutral midpoint at 0 (don’t know). This scale allows for interpreting negative ratings as indicating a degree of faking emotion and positive ratings a degree of genuine emotion. Previously, in Dawel et al. (2017) we used this task to evaluate the PoFA and a representative sample of front-facing female stimuli from the RaFD. We found most of these stimuli were perceived as *not* conveying genuine emotion, except for the PoFA happy expressions.

To assess perceptions of naturalness, we developed a new rating task. Natural expressions were defined as “*the types of expressions people show naturally in everyday life… can include expressions that people make on purpose, like smiling to be polite or to hide another feeling, as well as expressions that show how people really feel.”* Perceivers rated expression naturalness on a scale from −7 (completely unnatural) to +7 (completely natural), with a neutral midpoint at 0 (unsure). This scale allows for interpreting negative ratings as indicating a degree of unnaturalness and positive ratings a degree of naturalness.

**Fig. 2:**
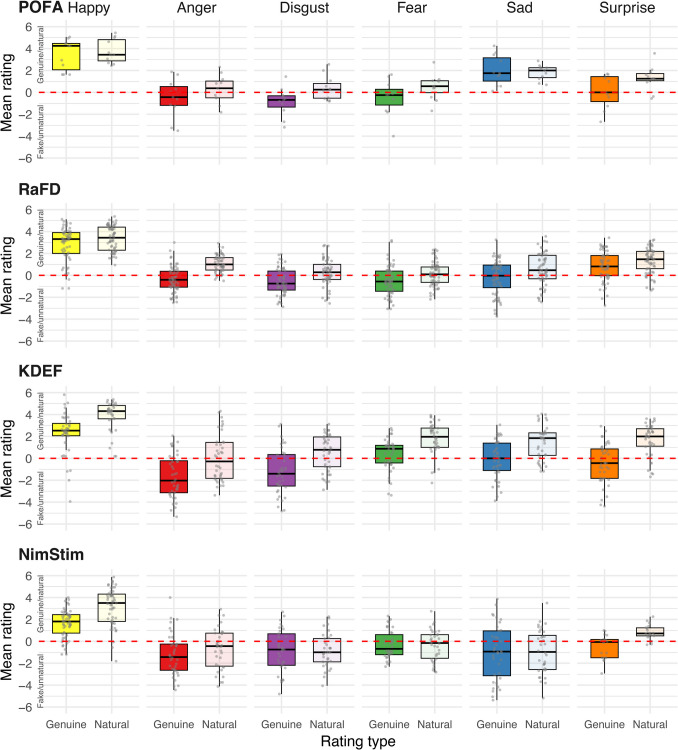
Mean Genuineness and Naturalness Ratings for Representative Samples of Stimuli from the Four Most Used (across 2000–2020, Dawel, Miller, et al., 2022) Facial Expression Databases. *Note.* Grey dots show mean ratings for individual stimuli

Results are presented in Table 2 and Fig. 2. For genuineness, more than half of the anger, disgust, and fear stimuli had mean ratings below zero, indicating they were perceived as faking emotion. This finding was consistent across all four databases, except that only 35% of KDEF fear expressions had mean ratings below zero. Large percentages of the sad and surprised stimuli were also rated below zero, except for the PoFA sad stimuli, which all had mean genuineness ratings above zero. The only category for which most stimuli were perceived as genuinely emotional was happiness, with stimuli from all four databases rarely being rated below zero. This finding for happy may reflect the high frequency and ease with which humans smile in everyday life (Calvo et al., 2014) and that the key muscles for smiling (zygomaticus major) have a high degree of voluntary control (although they are also innervated by the involuntary system; Hopf et al., 1992).

**Table 2:** Percentage of Stimuli with Mean Ratings Below Zero, Indicating Perceptions of Fakeness and Unnaturalness

% perceived as fake (*M* genuineness rating < 0)						% perceived as unnatural (*M* rating < 0)				
Emotion	PoFA	RaFD	KDEF	NimStim	*M*	PoFA	RaFD	KDEF	NimStim	*M*
Happy	0.0	5.0	10.0	13.2	7.1	0.0	0.0	0.0	5.7	1.4
Anger	54.5	63.3	75.0	77.8	67.7	45.5	6.7	50.0	63.9	41.5
Disgust	81.8	70.0	70.0	66.7	72.1	36.4	40.0	37.5	72.7	46.7
Fear	72.7	66.7	35.0	64.7	59.8	27.3	48.3	10.0	55.9	35.4
Sad	0.0	50.0	50.0	61.1	40.3	0.0	31.7	22.5	58.3	28.1
Surprise	45.5	25.0	57.5	50.0	44.5	18.2	13.3	15.0	11.1	14.4

*M* = average calculated across database percentage scores; *not* weighted by the number of stimuli per database

For naturalness, there was a strong correlation with mean genuineness across stimuli, *r* =.77, CI_95_ = [.74,.80], *p* <.001. However, the percentages of stimuli that had mean naturalness ratings less than zero tended to be lower, indicating that stimuli were less likely to be perceived as unnatural than as faking emotion. We confirmed stimulus-level mean ratings of naturalness were significantly higher than those for genuineness using ANOVA with rating type as the within-stimulus factor and database and emotion as between-stimulus factors, *F*(1, 852) = 352.0, *MSE* = 0.751, *p* <.001, η_p_^2^ =.29. This finding is consistent with our definition of natural expressions as including those that people make on purpose in natural social interactions, reflecting that some expressions not perceived as communicating genuine emotion can still look natural. Nevertheless, Table 2 shows that approximately one-third of all anger, disgust, fear and sad stimuli were perceived as unnatural.

Overall, these findings provide compelling evidence that standardized, posed expressions, which have historically dominated the literature, are often not perceived to be natural or conveying genuine internal emotion. Additionally, a confound exists between emotional valence and the perceived genuineness of expression stimuli in these databases. Among the six basic emotions typically represented, happiness is the only purely positive emotion (surprise can be positive or mildly negative; Noordewier & Breugelmans, 2013) *and* the only emotion for which most stimuli are perceived as expressing true emotion. This presents a methodological concern for studies reporting valence-based effects, as the perceived genuineness of expressions can influence participant responses (e.g., Gunnery & Ruben, 2015), potentially contributing to differences in findings for happy versus negatively-valenced expressions. Ekman (1980) himself highlighted this issue, noting that Sackeim et al.'s (1978) finding—where positive expressions were symmetrical and negative expressions asymmetrical—may not be attributable to valence. Instead, Ekman suggested that differences in stimulus creation methods might explain this pattern, as the happy expressions were captured during spontaneous interactions, whereas all negative expressions were posed.

## Why Has the Field Relied So Heavily on Standardized Posed Expressions?

The field’s past reliance on standardized posed expression databases stems from several factors; here, we highlight two key reasons. The first is the traditional emphasis in experimental research on maintaining rigorous control over stimulus features (Nastase et al., 2020). Standardizing various features of facial expression stimuli—including expression morphology, eye-gaze, head direction, and low-level attributes like lighting and resolution—ensures that only the intended factors, such as emotion category, influence participant responses in experimental studies. Historically, this approach was crucial, as accounting for variation in these factors statistically was challenging. However, advances in techniques like multilevel modelling (MLM), now allow researchers to control for extraneous variation statistically. Moreover, these statistical methods enable a more nuanced examination of target variables. For example, instead of comparing participants’ responses to broad categories of ‘happy’ and ‘sad’ expressions, researchers can analyse subtle variations in perceived valence at the individual stimuli level. Leveraging such sophisticated statistical approaches is essential for drawing robust conclusions from more variable stimulus sets that better capture the complexity and diversity of real-life facial displays.

The second reason is that, until recently, alternative types of stimuli were difficult to access. Following the development of BET, Ekman and colleagues were the first to widely distribute facial expression stimulus sets, contributing to their popularity: indeed, the PoFA set has been cited over 6000 times (Ekman & Friesen, 1976). Newer databases such as the KDEF (Lundqvist et al., 1998), NimStim (Tottenham et al., 2009), and RaFD (Langner et al., 2010) followed suit, emphasizing both accessibility and stimulus standardization. For decades, researchers had limited alternatives to these dominant stimuli, and most labs lacked the resources to create their own. However, social and technological advancements now provide easier access to a broad range of stimuli, including spontaneous and naturalistic expressions (Kim et al., 2025).

## Moving Forward: Different Types of Stimuli Produce Different Research Outcomes

Spontaneous and naturalistic expressions each offer different advantages to posed ones. Naturalistic expressions are typically considered to be ‘in-the-wild’ (Srinivasan & Martinez, 2018). They are not recorded in the lab but in real-world settings, and often it is unknown what the expressor is feeling. We distinguish naturalistic/in-the-wild expressions from spontaneous expressions which are recorded in the lab with an experimenter present and in circumstances where it is easier to know what emotion was elicited, because the emotion eliciting material is known and/or expressors provided self-reports. The fact that spontaneous expressions are recorded in the lab has technical advantages for ensuring high-quality stimuli through standardizing lighting and minimizing movement and facial occlusion (Gunes et al., 2008). On the other hand, naturalistic expressions recorded outside the lab present the types of challenges that people face in everyday interactions and, critically, include expressions that are organically posed for social reasons, such as polite smiling and masked expressions. In our own research, we have developed several new stimulus sets that demonstrate the importance and feasibility of both of these paths forward (Dawel et al., 2017; de la Harpe et al., 2024; Küster et al., 2021).

Critically, there are clear indications that using spontaneous and/or naturalistic expressions instead of posed expressions—particularly if the former are perceived as more genuinely emotional than the latter—can make a difference to research outcomes. One early indication that relying solely on posed expressions might be misleading comes from the late 1970s. LaRusso (1978) investigated the ability of individuals with paranoid schizophrenia and healthy controls to judge whether an expressor anticipated an electrical shock, comparing spontaneous and posed expression stimuli. The study produced the typical result for posed expressions, finding that individuals with schizophrenia were worse than controls at judging if the expressor anticipated being shocked. However, this pattern was reversed for spontaneous expressions, with individuals with schizophrenia performing better than controls. Similarly, Davis & Gibson (2000) found that people with paranoid schizophrenia were better at labelling negative emotions than controls for dynamic videos showing spontaneous expressions, but exhibited the typical deficit when the videos showed posed expressions.

Some of our own work has also directly compared responses to traditional posed stimuli (RaFD, KDEF), which were selected to be perceived as faking emotion, with responses to stimuli gathered from spontaneous and naturalistic sources, which were selected to be perceived as conveying genuine emotion and had high labelling agreement as per the posed expressions (Dawel et al. 2017).^3^ While these stimulus sets confound the creation method with perceived genuineness—posed expressions are perceived as fake, while spontaneous/naturalistic ones as genuine— evidence from these sets nevertheless illustrates that these intertwined factors can impact research outcomes. In one key example, we tested a longstanding theory predicting how the affective features associated with psychopathic traits arise (Dawel, Wright, et al., 2019). Critically, we found the predictions of this theory were supported only when tested with spontaneous/naturalistic expressions and not with posed ones. In another instance, we discovered that social anxiety symptoms were uniquely associated with reduced willingness to approach spontaneous/naturalistic smiling faces but not posed smilers (Dawel, Dumbleton, et al., 2019). Further investigation revealed that the differentiating factor was that spontaneous/naturalistic smilers were expected to want to interact for longer, exposing individuals with social anxiety to the potential for evaluation they fear. Finally, in Bothe et al. (2024) we found that associations between autistic traits and approach judgements were more pronounced when tested with spontaneous/naturalistic expressions than with posed ones.

There is also a large body of work testing people’s responses to Duchenne smiles—which are theorized to reflect genuine positive emotion—and non-Duchenne smiles, which are conceptualized as social (Ekman et al., 1990; for review see Krumhuber & Kappas, 2022). Compared to non-Duchenne smiles, Duchenne smiles are robustly found to elicit more positive and affiliative responses (Gunnery & Ruben, 2015), at least in White Western cultures (Thibault et al., 2012). The Duchenne marker also increase the perceptual salience and affective intensity of facial expressions (Malek et al., 2019; Miller et al., 2022). However, ironically, the majority of these studies have used posed Duchenne smiles (Gunnery & Ruben, 2015). Critical for the argument herein is that natural Duchenne smiles produce larger effects than do posed ones (Gunnery & Ruben, 2015).

We conjecture that one reason different stimulus types elicit different results is that perceiver’s affective reactions may be stronger when stimuli appear genuinely emotional, while the processing of posed expressions might rely more on cognitive channels. This possibility is alluded to by the influential SIMS model of smiling (Niedenthal et al., 2010), which proposes that humans connect with another’s emotional state through simulating their experience, including subtly mimicking their facial and other expressive displays. To illustrate this idea, consider how you might feel when interacting with a genuinely happy person compared to someone smiling politely at you. People tend to report a stronger affective response to the former, consistent with the idea of affective simulation. While evidence for stronger affective reactions to genuinely emotional over posed displays is scarce, some hints exist. Studies have found that facial mimicry is greater for Duchenne than non-Duchenne smiles (Rychlowska et al., 2014; cf. Krumhuber et al., 2014). McLellan et al. (2010) also found semantic priming was stronger for positive words preceded by spontaneous rather than posed happy expressions, and for negative words preceded by spontaneous rather than posed fearful expressions.

Ultimately, the evidence showing that participant responses differ between naturalistic, spontaneous expressions and posed ones highlights the need for conceptual replication of key findings in this field, ensuring that future research builds on a solid foundation. Using spontaneous and naturalistic expressions may also be increasingly important as the field moves toward using dynamic rather than static stimuli. For instance, the dynamic features of smiles play a critical role in genuineness perception (Ambadar et al., 2009; Krumhuber et al., 2013, 2023); and the perceptual discrimination of spontaneous from posed surprise expressions may be enhanced for dynamic relative to static stimuli (Zloteanu & Krumhuber, 2021).

## Recommendations for Advancing the Field

Table 3 proposes seven recommendations for advancing the field of face emotion research. Recommendation 1 comes directly from Fig. 1, proposing that future research should explicitly state whether it addresses the processes of (Q1) expression production, (Q2) perceptual accuracy, or Q3) perception/responding; and also that past research should be interpreted in relation to the distinction between these three question types. We anticipate this disentangling will assist with interpreting and harmonizing the literature, as well as highlight the imbalance between the large number of studies addressing Q3 and relative lack of evidence concerning Q2. We believe the explicit separation of perception (Q3) from perceptual accuracy (Q2) is particularly important for moving the field forward. For example, the reconceptualization of perception as a measure of consensus points to novel theoretical interpretations of past work and new methods. Researchers could adopt metrics from facial impressions research, such as intraclass correlation coefficients (ICCs), to measure consensus perceptions (see Hehman et al., 2017). This approach will enable a more precise understanding of how individuals interpret facial expressions collectively, advancing our grasp of the relative importance of perceiver and stimulus contributions to expression perception.

**Table 3:** Recommendations

R1. Past literature should be interpreted in relation to the three question types set out in Fig. 1, and future studies should explicitly state if they address the processes of (Q1) expression production, (Q2) perceptual accuracy, or (Q3) perception/responding.	
R2. The development of novel naturalistic and spontaneous stimulus sets is critical, and offers opportunity for increasing demographic diversity, which is also traditionally lacking in expression research.	
R3. Stimulus metadata should include measures of expressors’ internal emotion, such as physiological and self-report data, where possible (e.g., for spontaneous expressions captured in the lab); this is critical for enabling tests of Q1 and Q2. R4. Stimulus metadata should include perceiver ratings of genuineness and naturalness (in addition to perceiver emotion labelling and intensity and valence rating data), so that these variables can be used to select stimuli (R5) and/or model their effects in analysis (R6).	
R5. Stimulus selection should be carefully matched to the research question, and the rationale for selection explicitly reported in the study methods.	
R6. As an alternative to experimentally controlling target variables through stimulus selection (which can be more difficult for naturalistic expressions), sophisticated analytical approaches such as MLM offer a valuable tool for examining the effects of naturally occurring differences in target variables (e.g., assessing how perceived genuineness influences participant responses) and/or parsing out the effects of extraneous variables (e.g., natural variation in viewpoint).	
R7. Key studies—particularly those that are foundational for the field moving forward—would benefit from conceptual replication with more ecologically valid stimuli (spontaneous and/or naturalistic expressions as appropriate).	

Recommendation 2 emphasizes the need for expression stimuli to capture real-life facial behavior, with greater demographic diversity. For findings to be generalizable to real-world interactions, stimuli must reflect the wide range of facial expressions encountered in everyday life—what we refer to as the “face diet”. Improving the demographic diversity of stimuli will also open opportunities to understand cross-cultural and individual differences in expressive displays (Q1), and how these factors influence perceptual accuracy (Q2) and perceptual responses (Q3). For example, it may be the case that naturally-occurring expressions are not observed for some emotions in some cultures because they are not readily communicated through the face.

Recommendations 3 and 4 call for expanding the types of metadata collected on expression stimuli. In addition to traditional measures such as perceiver emotion labelling and intensity and valence ratings, the field would benefit from databases that include the expressors’ internal emotions, as is critical for addressing Q1 and Q2, and perceiver data on other attributes that might impact other responses measured for Q3, such as how natural or genuine the physical expression looks. Note, we do not propose using ratings of emotional genuineness or naturalness for validation purposes—validity should be determined by how expressions are elicited and measures of expressors’ internal experience. Instead, these ratings could be used to select stimuli for experimental designs that aim to manipulate these variables or to model their effects in statistical analyses (recommendation 6).

Recommendation 5 is that researchers carefully consider the selection of expression stimuli and provide a clear rationale for their choices. While we advocate for the use of spontaneous and naturalistic expressions, posed expressions remain valuable in specific contexts—where high experimental control is essential, or perhaps where questions concern visual/cognitive processes rather than affective ones. Each type of stimulus offers distinct strengths. Spontaneous expressions can be captured with high-quality equipment and standardized elicitation, and it is usually possible to obtain self-report and other measures of internal emotion (recommendation 3). However, it may be difficult to elicit certain types of spontaneous expressions for practical and ethical reasons. For example, it is not ethically responsible to elicit intense terror in the lab. Naturalistic stimuli, while less controlled than spontaneous ones, provide a more accurate reflection of everyday interactions, and may capture a broader range of facial behavior. These types of stimuli could open the door to new insights into the natural variation in people’s expressive patterns. For example, it may be that some expressive patterns are extremely rare in the modern social world, or that this variation differs in important ways across cultures. However, difficulty in obtaining self-reports of emotion and contextual information, along with variations in viewpoint, lighting and other visual features, may make it difficult to pinpoint underlying mechanisms. Researchers should carefully balance these pros and cons against their research question and look for opportunities to obtain convergent evidence using different stimulus types and methods.

Recommendation 6 is the adoption of alternative analytical approaches to better model the complexity of facial expressions. Shifts in the accessibility of advanced analytic approaches, such as multilevel modelling (MLM), mean that researchers can now statistically model the effects of variables of interest (e.g., perceived intensity or genuineness) or parse out unwanted extraneous variation (e.g., face viewpoint) for each individual stimulus, rather than having to control for these factors in the experimental design. This shift is important because part of the rationale for standardizing traditional posed stimuli is that it controlled for extraneous factors that might influence results. This noise can now be parsed out post- rather than pre-experiment, through statistical modelling rather than stimulus selection respectively. Thus any ‘noise’ that is introduced through the complex and nuanced appearance of naturalistic expressions can be managed analytically.

Finally, recommendation 7 highlights the need for replicative and convergence-testing studies, especially given the recent replication crisis (Derksen & Morawski, 2022). A systematic effort to replicate key findings in face perception research would serve two important functions. First, to confirm which aspects of the current knowledge base are robust. Second, to evaluate the generalizability of findings based on posed expressions to more naturalistic and spontaneous facial behavior. Rather than directly repeating past studies, conceptual replications that employ contemporary stimuli and methods are likely to provide more meaningful insights. It is also important to recognize that some findings from posed expressions, particularly in the realm of visual/cognitive processes, may be reliable and should not be discarded without due consideration (Talipski et al., 2025).

## Conclusion

The field must prioritize building a knowledge base that is both generalizable to real-world contexts and capable of elucidating the underlying mechanisms of facial expression, leveraging the tools available for experimental and analytic control. A multi-method approach, combining controlled stimuli and experimental paradigms with real-world observational studies of naturalistic facial behaviour, will provide more robust evidence through seeking empirical convergence. Note, it is critical that the approach use actual human behaviour. Researchers may be tempted to generate face stimuli using artificial intelligence (AI), given the high level of realism these have achieved (Miller, Steward, et al., 2023; Nightingale & Farid, 2022). However—despite their apparent realism—it is clear these images do not reflect the real-world human ‘face diet’ and are plagued by racial and other biases (Gusdorff et al., 2024). Thus the use of AI-generated faces has potential to distort scientific outcomes if applied as stand-ins for real human faces (Miller, Foo, et al., 2023). Emphasizing the study of real-world human behaviour and implementing these seven recommendations should help ensure that future research is both ecologically valid and methodologically robust, driving deeper theoretical insights and practical advancements.

## Footnotes

1 A smile characterized by “crow’s feet” wrinkles around the eyes (Ekman et al., 1990), which is theorized to signify genuinely felt happiness (Frank & Ekman, 1993).

2 While some argue that configuring one’s face in certain ways can induce an internal emotional experience (i.e., the ‘facial feedback hypothesis’; Levenson et al., 1990), the expressions captured in the most commonly used stimuli precede (rather than responsively express) any associated internal emotion. Moreover, this internal experience is likely weak relative to the outward, highly-intense posed facial display.

3 Just as we argue that consensus on emotion labelling alone is an insufficient criterion for stimulus validity, we also contend that consensus on perceived genuineness is neither necessary nor sufficient to establish the validity of spontaneous and naturalistic expressions. However, a key aim in developing these stimulus sets was to determine whether perceived genuineness influences research outcomes in studies addressing Q3 (other types of perception and responses). Critical for this purpose, the perceived-as-genuine and perceived-as-posed sets were carefully matched within each emotion category for factors like expression intensity, face sex, and viewpoint.

## Additional Information

### Data Availability

The full face rating method, data, and analysis code are available at: https://osf.io/gtnx5/?view_only=0451d7def4864b558ad261db56fd11ab.

## References

[CR1] Adams, R. B., Albohn, D. N., & Kveraga, K. (2017). Social vision: Applying a social-functional approach to face and expression perception. *Current Directions in Psychological Science, 26*(3), 243–248. 10.1177/0963721417706392 29606807 10.1177/0963721417706392 PMC5873322

[CR2] Adolphs, R., Mlodinow, L., & Barrett, L. F. (2019). What is an emotion? *Current Biology, 29*(20), R1060–R1064. 10.1016/j.cub.2019.09.008 31639344 10.1016/j.cub.2019.09.008 PMC7749626

[CR3] Ambadar, Z., Cohn, J. F., & Reed, L. I. (2009). All smiles are not created equal: Morphology and timing of smiles perceived as amused, polite, and embarrassed/nervous. *Journal of Nonverbal Behavior, 33*(1), 17–34. 10.1007/s10919-008-0059-5 19554208 10.1007/s10919-008-0059-5 PMC2701206

[CR4] Ansfield, M. E. (2007). Smiling when distressed: When a smile is a frown turned upside down. *Personality and Social Psychology Bulletin, 33*(6), 763–775. 10.1177/0146167206297398 17483396 10.1177/0146167206297398

[CR5] Aviezer, H., Ensenberg, N., & Hassin, R. R. (2017). The inherently contextualized nature of facial emotion perception. *Current Opinion in Psychology, 17*, 47–54. 10.1016/j.copsyc.2017.06.006 28950972 10.1016/j.copsyc.2017.06.006

[CR6] Aviezer, H., Trope, Y., & Todorov, A. (2012). Body cues, not facial expressions, discriminate between intense positive and negative emotions. *Science, 338*(6111), 1225–1229. 10.1126/science.1224313 23197536 10.1126/science.1224313

[CR7] Baek, E. C., & Parkinson, C. (2022). Shared understanding and social connection: Integrating approaches from social psychology, social network analysis, and neuroscience. *Social and Personality Psychology Compass, 16*(11), e12710. 10.1111/spc3.12710 36582415 10.1111/spc3.12710 PMC9786704

[CR8] Barrett, L. F. (2017). The theory of constructed emotion: An active inference account of interoception and categorization. *Social Cognitive and Affective Neuroscience,**12*(1), 1–23. 10.1093/scan/nsw154 27798257 10.1093/scan/nsw154 PMC5390700

[CR9] Barrett, L. F., Adolphs, R., Marsella, S., Martinez, A. M., & Pollak, S. D. (2019). Emotional expressions reconsidered: Challenges to inferring emotion from human facial movements. *Psychological Science in the Public Interest, 20*(1), 1–68. 10.1177/1529100619832930 31313636 10.1177/1529100619832930 PMC6640856

[CR10] Bothe, E., Jeffery, L., Dawel, A., Donatti-Liddelow, B., & Palermo, R. (2024). Autistic traits are associated with differences in the perception of genuineness and approachability in emotional facial expressions, independently of alexithymia. *Emotion, 24*(5), 1322–1337. 10.1037/emo0001350 38421790 10.1037/emo0001350

[CR11] Butler, E. A., Egloff, B., Wilhelm, F. H., Smith, N. C., Erickson, E. A., & Gross, J. J. (2003). The social consequences of expressive suppression. *Emotion, 3*(1), 48–67. 10.1037/1528-3542.3.1.48 12899316 10.1037/1528-3542.3.1.48

[CR12] Calvo, M. G., Gutiérrez-García, A., Fernández-Martín, A., & Nummenmaa, L. (2014). Recognition of facial expressions of emotion is related to their frequency in everyday life. *Journal of Nonverbal Behavior, 38*(4), 549–567. 10.1007/s10919-014-0191-3

[CR13] Cohn, J. F., Schmidt, K., Gross, R., & Ekman, P. (2002). Individual differences in facial expression: Stability over time, relation to self-reported emotion, and ability to inform person identification. In *Proceedings. Fourth IEEE international conference on multimodal interfaces* (pp. 491–496). 10.1109/ICMI.2002.1167045

[CR14] Cohn, J. F., & Schmidt, K. L. (2004). The timing of facial motion in posed and spontaneous smiles. *International Journal of Wavelets, Multiresolution and Information Processing,**2*(2), 121–132. 10.1142/S021969130400041X

[CR15] Crivelli, C., & Fridlund, A. J. (2018). Facial displays are tools for social influence. *Trends in Cognitive Sciences, 22*(5), 388–399. 10.1016/j.tics.2018.02.006 29544997 10.1016/j.tics.2018.02.006

[CR16] Davidson, R. J., Ekman, P., Saron, C. D., Senulis, J. A., & Friesen, W. V. (1990). Approach-withdrawal and cerebral asymmetry: Emotional expression and brain physiology: I. *Journal of Personality and Social Psychology,**58*(2), 330–341. 10.1037/0022-3514.58.2.330 2319445

[CR17] Davis, P. J., & Gibson, M. G. (2000). Recognition of posed and genuine facial expressions of emotion in paranoid and nonparanoid schizophrenia. *Journal of Abnormal Psychology, 109*(3), 445–450. 10.1037/0021-843X.109.3.445 11016114

[CR18] Dawel, A., O’Kearney, R., McKone, E., & Palermo, R. (2012). Not just fear and sadness: Meta-analytic evidence of pervasive emotion recognition deficits for facial and vocal expressions in psychopathy. *Neuroscience and Biobehavioral Reviews, 36*(10), 2288–2304. 10.1016/j.neubiorev.2012.08.006 22944264 10.1016/j.neubiorev.2012.08.006

[CR19] Dawel, A., Wright, L., Irons, J., Dumbleton, R., Palermo, R., O’Kearney, R., & McKone, E. (2017). Perceived emotion genuineness: Normative ratings for popular facial expression stimuli and the development of perceived-as-genuine and perceived-as-fake sets. *Behavior Research Methods, 49*(4), 1539–1562. 10.3758/s13428-016-0813-2 27928745 10.3758/s13428-016-0813-2

[CR20] Dawel, A., Dumbleton, R., O’Kearney, R., Wright, L., & McKone, E. (2019). Reduced willingness to approach genuine smilers in social anxiety explained by potential for social evaluation, not misperception of smile authenticity. *Cognition and Emotion, 33*(7), 1342–1355. 10.1080/02699931.2018.1561421 30585120 10.1080/02699931.2018.1561421

[CR21] Dawel, A., Wright, L., Dumbleton, R., & McKone, E. (2019). All tears are crocodile tears: Impaired perception of emotion authenticity in psychopathic traits. *Personality Disorders: Theory, Research, and Treatment, 10*(2), 185–197. 10.1037/per0000301 10.1037/per0000301 30010374

[CR22] Dawel, A., Miller, E. J., Horsburgh, A., & Ford, P. (2022). A systematic survey of face stimuli used in psychological research 2000–2020. *Behavior Research Methods, 54*(4), 1889–1901. 10.3758/s13428-021-01705-3 34731426 10.3758/s13428-021-01705-3

[CR23] Dawel, A., Ashhurst, C., & Monaghan, C. (2023). A three-dimensional model of emotional display rules: Model invariance, external validity, and gender differences. *Emotion, 23*(5), 1410–1422. 10.1037/emo0001176 36227313 10.1037/emo0001176

[CR24] de la Harpe, S., Palermo, R., Brown, E., Fay, N., & Dawel, A. (2024). People attribute a range of highly-varied and socially-bound meanings to naturalistic sad facial expressions. *Journal of Nonverbal Behavior,**48*(3), 465–493. 10.1007/s10919-024-00463-y

[CR25] DeBruine, L. M., & Jones, B. (2022). *Determining the number of raters for reliable mean ratings*. 10.17605/OSF.IO/X7FUS

[CR26] Derksen, M., & Morawski, J. (2022). Kinds of replication: Examining the meanings of “conceptual replication” and “direct replication.” *Perspectives on Psychological Science,**17*(5), 1490–1505. 10.1177/17456916211041116 35245130 10.1177/17456916211041116 PMC9442273

[CR27] Douglas, K. M., Porter, R. J., & Johnston, L. (2012). Sensitivity to posed and genuine facial expressions of emotion in severe depression. *Psychiatry Research, 196*(1), 72–78. 10.1016/j.psychres.2011.10.019 22370153 10.1016/j.psychres.2011.10.019

[CR28] Durán, J. I., & Fernández-Dols, J.-M. (2021). Do emotions result in their predicted facial expressions? A meta-analysis of studies on the co-occurrence of expression and emotion. *Emotion, 21*(7), 1550–1569. 10.1037/emo0001015 34780241 10.1037/emo0001015

[CR29] Durán, J. I., & Fernández-Dols, J.-M. (2023). Basic emotions do not reliably co-occur with predicted facial expressions: Reply to Witkower et al. *Emotion, 23*(3), 908–910. 10.1037/emo0001227 37079838 10.1037/emo0001227

[CR30] Ekman, P. (1980). Asymmetry in facial expression. *Science, 209*, 833–834. 7403851 10.1126/science.7403851

[CR31] Ekman, P. (1992). Are there basic emotions? *Psychological Review,**99*(3), 550–553. 10.1037/0033-295X.99.3.550 1344638 10.1037/0033-295x.99.3.550

[CR32] Ekman, P. (1994). Strong evidence for universals in facial expressions: A reply to Russell’s mistaken critique. *Psychological Bulletin,**115*(2), 268–287. 10.1037/0033-2909.115.2.268 8165272 10.1037/0033-2909.115.2.268

[CR33] Ekman, P. (2007). The directed facial action task: Emotional responses without appraisal. In J. A. Coan & J. J. B. Allen (Eds.), *Handbook of emotional elicitation and assessment* (pp. 47–53). Oxford University Press.

[CR34] Ekman, P., & Cordaro, D. (2011). What is meant by calling emotions basic. *Emotion Review, 3*(4), 364–370. 10.1177/1754073911410740

[CR35] Ekman, P., Davidson, R. J., & Friesen, W. V. (1990). The Duchenne smile: Emotional expression and brain physiology: II. *Journal of Personality and Social Psychology,**58*(2), 342–353. 10.1037/0022-3514.58.2.342 2319446

[CR36] Ekman, P., & Friesen, W. V. (1976). *Pictures of facial affect* (pp. 1–8). Consulting Psychologists Press.

[CR37] Ekman, P., Sorenson, E. R., & Friesen, W. V. (1969). Pan-cultural elements in facial displays of emotion. *Science,**164*(3875), 86–88. 10.1126/science.164.3875.86 5773719 10.1126/science.164.3875.86

[CR38] Elfenbein, H. A., & Ambady, N. (2003). Universals and Cultural Differences in Recognizing Emotions. *Current Directions in Psychological Science,**12*(5), 159–164. 10.1111/1467-8721.01252

[CR39] Fernández-Dols, J.-M., & Ruiz-Belda, M.-A. (1995). Are smiles a sign of happiness? Gold medal winners at the olympic games. *Journal of Personality and Social Psychology, 69*(6), 1113–1119. 10.1037/0022-3514.69.6.1113

[CR40] Fischer, A. H., Manstead, A. S. R., & Zaalberg, R. (2003). Social influences on the emotion process. *European Review of Social Psychology, 14*(1), 171–201. 10.1080/10463280340000054

[CR41] Foo, Y. Z., Sutherland, C. A. M., Burton, N. S., Nakagawa, S., & Rhodes, G. (2022). Accuracy in facial trustworthiness impressions: Kernel of truth or modern physiognomy? A meta-analysis. *Personality and Social Psychology Bulletin, 48*(11), 1580–1596. 10.1177/01461672211048110 34609231 10.1177/01461672211048110

[CR42] Frank, M. G., & Ekman, P. (1993). Not all smiles are created equal: The differences between enjoyment and nonenjoyment smiles. *Humor, 6*(1), 9–26. 10.1515/humr.1993.6.1.9

[CR43] Fridlund, A. J. (1991). Sociality of solitary smiling: Potentiation by an implicit audience. *Journal of Personality and Social Psychology, 60*(2), 229–240. 10.1037/0022-3514.60.2.229

[CR44] Gunes, H., Piccardi, M., & Pantic, M. (2008). From the lab to the real world: Affect recognition using multiple cues and modalities. In J. Or (Ed.), *Affective computing: Focus on emotion expression, synthesis, and recognition* (pp. 185–218). I-Tech Education and Publishing. 10.5772/6180

[CR45] Gunnery, S. D., & Ruben, M. A. (2015). Perceptions of Duchenne and non-Duchenne smiles: A meta-analysis. *Cognition and Emotion,**30*(3), 501–515. 10.1080/02699931.2015.1018817 25787714 10.1080/02699931.2015.1018817

[CR46] Gusdorff, M., Grissom, A., Rocha Neto, J. F. S., Lin, Y., Trotter, R., & Lei, R. F. (2024). Considering how machine-learning algorithms (re)produce social biases in generated faces. *Social and Personality Psychology Compass, 18*(11), e70021. 10.1111/spc3.70021

[CR47] Haile, J. C., Palermo, R., Dawel, A., Krumhuber, E. G., Sutherland, C. A. M., & Bell, J. (under review). *Eye Believe You: Gaze direction affects the perceived believability of facial expressions displayed by computer-generated people.* 10.1080/02699931.2026.2620987 41592021

[CR48] Hall, J. A., & Horgan, T. G. (2003). Happy affect and smiling: Is their relation moderated by interpersonal power? *Emotion, 3*(3), 303–309. 10.1037/1528-3542.3.3.303 14498798 10.1037/1528-3542.3.3.303

[CR49] Hedgecoth, N., Strand, N., & Adams, R. B. (2023). The intersection of race, gender/sex, and age in emotion perception from faces and bodies. In U. Hess, R. B. Adams Jr., & R. E. E. Kleck (Eds.), *Emotion communication by the aging face and body: A multidisciplinary view* (pp. 106–139). Cambridge University Press.

[CR50] Hehman, E., Sutherland, C. A. M., Flake, J. K., & Slepian, M. L. (2017). The unique contributions of perceiver and target characteristics in person perception. *Journal of Personality and Social Psychology, 113*(4), 513–529. 10.1037/pspa0000090 28481616 10.1037/pspa0000090

[CR51] Henrich, J., Heine, S. J., & Norenzayan, A. (2010). The weirdest people in the world? *Behavioral and Brain Sciences,**33*(2–3), 61–83. 10.1017/S0140525X0999152X 20550733 10.1017/S0140525X0999152X

[CR52] Hopf, H. C., Muller-Forell, W., & Hopf, N. J. (1992). Localization of emotional and volitional facial paresis. *Neurology, 42*(10), 1918–1918. 10.1212/WNL.42.10.1918 1407573 10.1212/wnl.42.10.1918

[CR53] Jack, R. E., Garrod, O. G. B., Yu, H., Caldara, R., & Schyns, P. G. (2012). Facial expressions of emotion are not culturally universal. *Proceedings of the National Academy of Sciences, 109*(19), 7241–7244. 10.1073/pnas.1200155109 10.1073/pnas.1200155109 PMC3358835 22509011

[CR54] Jack, R. E., & Schyns, P. G. (2015). The human face as a dynamic tool for social communication. *Current Biology, 25*(14), R621–R634. 10.1016/j.cub.2015.05.052 26196493 10.1016/j.cub.2015.05.052

[CR55] Jaeger, B., Oud, B., Williams, T., Krumhuber, E. G., Fehr, E., & Engelmann, J. B. (2022). Can people detect the trustworthiness of strangers based on their facial appearance? *Evolution and Human Behavior, 43*(4), 296–303. 10.1016/j.evolhumbehav.2022.04.004

[CR56] Kim, H., Bian, Y., & Krumhuber, E. G. (2025). A review of 25 spontaneous and dynamic facial expression databases of basic emotions. *Affective Science,**6*, 380–394. 10.1007/s42761-024-00289-3 40605939 10.1007/s42761-024-00289-3 PMC12209106

[CR57] Kret, M. E. (2015). Emotional expressions beyond facial muscle actions. A call for studying autonomic signals and their impact on social perception. *Frontiers in Psychology, 6*, 1–10. 10.3389/fpsyg.2015.00711 26074855 10.3389/fpsyg.2015.00711 PMC4443639

[CR58] Krumhuber, E. G., & Kappas, A. (2022). More what Duchenne smiles do, less what they express. *Perspectives on Psychological Science, 17*(6), 1566–1575. 10.1177/17456916211071083 35712993 10.1177/17456916211071083

[CR59] Krumhuber, E. G., Kappas, A., & Manstead, A. S. R. (2013). Effects of dynamic aspects of facial expressions: A review. *Emotion Review, 5*(1), 41–46. 10.1177/1754073912451349

[CR60] Krumhuber, E. G., Likowski, K. U., & Weyers, P. (2014). Facial mimicry of spontaneous and deliberate Duchenne and non-Duchenne smiles. *Journal of Nonverbal Behavior, 38*(1), 1–11. 10.1007/s10919-013-0167-8

[CR61] Krumhuber, E. G., & Manstead, A. S. R. (2009). Can Duchenne smiles be feigned? New evidence on felt and false smiles. *Emotion, 9*(6), 807–820. 10.1037/a0017844 20001124 10.1037/a0017844

[CR62] Krumhuber, E. G., Skora, L. I., Hill, H. C. H., & Lander, K. (2023). The role of facial movements in emotion recognition. *Nature Reviews Psychology, 2*(5), 283–296. 10.1038/s44159-023-00172-1

[CR63] Krumhuber, E. G., Skora, L., Küster, D., & Fou, L. (2016). A review of dynamic datasets for facial expression research. *Emotion Review,**9*(3), 280–292. 10.1177/1754073916670022

[CR64] Kuppens, P., & Tong, E. M. W. (2010). An appraisal account of individual differences in emotional experience: Individual differences in emotional experience. *Social and Personality Psychology Compass, 4*(12), 1138–1150. 10.1111/j.1751-9004.2010.00324.x

[CR65] Küster, D., Baker, M., & Krumhuber, E. G. (2021). PDSTD - the Portsmouth dynamic spontaneous tears database. *Behavior Research Methods,**54*, 2678–2692. 10.3758/s13428-021-01752-w 34918224 10.3758/s13428-021-01752-w PMC9729121

[CR66] Küster, D., Steinert, L., Baker, M., Bhardwaj, N., & Krumhuber, E. G. (2023). Teardrops on my face: Automatic weeping detection from nonverbal behavior. *IEEE Transactions on Affective Computing, 14*(4), 3001–3012. 10.1109/TAFFC.2022.3228749

[CR67] Langner, O., Dotsch, R., Bijlstra, G., Wigboldus, D. H. J., Hawk, S. T., & van Knippenberg, A. (2010). Presentation and validation of the Radboud faces database. *Cognition & Emotion, 24*(8), 1377–1388. 10.1080/02699930903485076

[CR68] LaRusso, L. (1978). Sensitivity of paranoid patients to nonverbal cues. *Journal of Abnormal Psychology,**87*(5), 463–471. 10.1037/0021-843X.87.5.463 701598 10.1037//0021-843x.87.5.463

[CR69] Levenson, R. W., Ekman, P., & Friesen, W. V. (1990). Voluntary facial action generates emotion-specific autonomic nervous system activity. *Psychophysiology,**27*(4), 363–384. 10.1111/j.1469-8986.1990.tb02330.x 2236440 10.1111/j.1469-8986.1990.tb02330.x

[CR70] Lloyd, E. P., & Summers, K. M. (n.d.). Pain expression and reception as intergroup phenomena. *Affective Science* Special Issue on Face Emotion. 10.1007/s42761-025-00349-2 PMC13003710 41868984

[CR71] Luminet, O., Nielson, K. A., & Ridout, N. (2021). Cognitive-emotional processing in alexithymia: An integrative review. *Cognition and Emotion, 35*(3), 449–487. 10.1080/02699931.2021.1908231 33787442 10.1080/02699931.2021.1908231

[CR72] Lundqvist, D., Flykt, A., & Öhman, A. (1998). *The Karolinska directed emotional faces—KDEF (CD-ROM)*. Department of Clinical Neuroscience, Psychology section, Karolinska Institutet.

[CR73] Malek, N., Messinger, D., Gao, A. Y. L., Krumhuber, E., Mattson, W., Joober, R., Tabbane, K., & Martinez-Trujillo, J. C. (2019). Generalizing Duchenne to sad expressions with binocular rivalry and perception ratings. *Emotion, 19*(2), 234–241. 10.1037/emo0000410 29888933 10.1037/emo0000410

[CR74] Matsumoto, D., & Ekman, P. (1988). *Japanese and Caucasian facial expressions of emotion (JACFEE)*. Intercultural and Emotion Research Laboratory, Department of Psychology, San Francisco State University.

[CR75] Matsumoto, D., & Willingham, B. (2006). The thrill of victory and the agony of defeat: Spontaneous expressions of medal winners of the 2004 Athens Olympic games. *Journal of Personality and Social Psychology, 91*(3), 568–581. 10.1037/0022-3514.91.3.568 16938038 10.1037/0022-3514.91.3.568

[CR76] Matsumoto, D., & Wilson, M. (n.d.). What’s in a facial stimulus? *Affective Science, Special Issue on Face Emotion*. 10.1007/s42761-025-00342-9 PMC13086986 42006836

[CR77] Matsumoto, D., Yoo, S. H., Fontaine, J., Anguas-Wong, A. M., Arriola, M., Ataca, B., Bond, M. H., Boratav, H. B., Breugelmans, S. M., Cabecinhas, R., Chae, J., Chin, W. H., Comunian, A. L., Degere, D. N., Djunaidi, A., Fok, H. K., Friedlmeier, W., Ghosh, A., Glamcevski, M., et al. (2008). Mapping expressive differences around the world: The relationship between emotional display rules and individualism versus collectivism. *Journal of Cross-Cultural Psychology, 39*(1), 55–74. 10.1177/0022022107311854

[CR78] McLellan, T. L., Johnston, L., Dalrymple-Alford, J., & Porter, R. (2010). Sensitivity to genuine versus posed emotion specified in facial displays. *Cognition & Emotion, 24*(8), 1277–1292. 10.1080/02699930903306181

[CR79] McLellan, T., & McKinlay, A. (2013). Sensitivity to emotion, empathy and theory of mind: Adult performance following childhood TBI. *Brain Injury,**27*(9), 1032–1037. 10.3109/02699052.2013.794965 23781878 10.3109/02699052.2013.794965

[CR80] Mewton, P., Dawel, A., Miller, E. J., Shou, Y., & Christensen, B. K. (2025). Meta-analysis of face perception in schizophrenia spectrum disorders: Evidence for differential impairment in emotion face perception. *Schizophrenia Bulletin,**51*(1), 17–36. 10.1093/schbul/sbae130 10.1093/schbul/sbae130 PMC11661959 39136259

[CR81] Miles, L. K., & Johnston, L. (2007). Detecting happiness: Perceiver sensitivity to enjoyment and non-enjoyment smiles. *Journal of Nonverbal Behavior, 31*(4), 259–275. 10.1007/s10919-007-0036-4

[CR82] Miller, E. J., Krumhuber, E. G., & Dawel, A. (2022). Observers perceive the duchenne marker as signaling only intensity for sad expressions, not genuine emotion. *Emotion, 22*(5), 907–919. 10.1037/emo0000772 32718174 10.1037/emo0000772

[CR83] Miller, E. J., Foo, Y. Z., Mewton, P., & Dawel, A. (2023). How do people respond to computer-generated versus human faces? A systematic review and metaanalyses. *Computers in Human Behavior Reports,**10*, 100283. 10.1016/j.chbr.2023.100283

[CR84] Miller, E. J., Steward, B. A., Witkower, Z., Sutherland, C. A. M., Krumhuber, E. G., & Dawel, A. (2023). AI hyperrealism: Why AI faces are perceived as more real than human ones. *Psychological Science, 34*(12), 1390–1403. 10.1177/09567976231207095 37955384 10.1177/09567976231207095

[CR85] Monaghan, C., Shou, Y., Mewton, P., Quayle, A., & Dawel, A. (2025). The expression regulation scale (ERS): Validation of three emotion domains for expressive norms with close and distant others in private and public situations. *Assessment*. 10.1177/10731911251333664 10.1177/10731911251333664 PMC12924882 40326386

[CR86] Nastase, S. A., Goldstein, A., & Hasson, U. (2020). Keep it real: Rethinking the primacy of experimental control in cognitive neuroscience. *NeuroImage, 222*, 117254. 10.1016/j.neuroimage.2020.117254 32800992 10.1016/j.neuroimage.2020.117254 PMC7789034

[CR87] Niedenthal, P. M., Mermillod, M., Maringer, M., & Hess, U. (2010). The simulation of smiles (SIMS) model: Embodied simulation and the meaning of facial expression. *Behavioral and Brain Sciences, 33*, 417–433; discussion 433-480. 10.1017/S0140525X10000865 21211115 10.1017/S0140525X10000865

[CR88] Nightingale, S. J., & Farid, H. (2022). AI-synthesized faces are indistinguishable from real faces and more trustworthy. *Proceedings of the National Academy of Sciences, 119*(8), e2120481119. 10.1073/pnas.2120481119 10.1073/pnas.2120481119 PMC8872790 35165187

[CR89] Noordewier, M. K., & Breugelmans, S. M. (2013). On the valence of surprise. *Cognition and Emotion, 27*(7), 1326–1334. 10.1080/02699931.2013.777660 23560688 10.1080/02699931.2013.777660

[CR90] Olivola, C. Y., Funk, F., & Todorov, A. (2014). Social attributions from faces bias human choices. *Trends in Cognitive Sciences, 18*(11), 566–570. 10.1016/j.tics.2014.09.007 25344029 10.1016/j.tics.2014.09.007

[CR91] Palermo, R., Jeffery, L., Lewandowsky, J., Fiorentini, C., Irons, J. L., Dawel, A., Burton, N., McKone, E., & Rhodes, G. (2018). Adaptive face coding contributes to individual differences in facial expression recognition independently of affective factors. *Journal of Experimental Psychology: Human Perception and Performance, 44*(4), 503–517. 10.1037/xhp0000463 28825500 10.1037/xhp0000463

[CR92] Palermo, R., O’Connor, K. B., Davis, J. M., Irons, J., & McKone, E. (2013). New tests to measure individual differences in matching and labelling facial expressions of emotion, and their association with ability to recognise vocal emotions and facial identity. *PLoS One, 8*(6), e68126. 10.1371/journal.pone.0068126 23840821 10.1371/journal.pone.0068126 PMC3695959

[CR93] Rinn, W. E. (1984). The neuropsychology of facial expression: A review of the neurological and psychological mechanisms for producing facial expressions. *Psychological Bulletin, 95*(1), 52–77. 10.1037/0033-2909.95.1.52 6242437

[CR94] Ruiz-Belda, M.-A., Fernández-Dols, J.-M., Carrera, P., & Barchard, K. (2003). Spontaneous facial expressions of happy bowlers and soccer fans. *Cognition & Emotion, 17*(2), 315–326. 10.1080/02699930302288 29715720 10.1080/02699930302288

[CR95] Russell, J. A. (1994). Is there universal recognition of emotion from facial expression? A review of the cross-cultural studies. *Psychological Bulletin, 115*(1), 102–141. 8202574 10.1037/0033-2909.115.1.102

[CR96] Rychlowska, M., Cañadas, E., Wood, A., Krumhuber, E. G., Fischer, A., & Niedenthal, P. M. (2014). Blocking mimicry makes true and false smiles look the same. *PLoS One, 9*(3), e90876. 10.1371/journal.pone.0090876 24670316 10.1371/journal.pone.0090876 PMC3966726

[CR97] Sackeim, H. A., Gur, R. C., & Saucy, M. C. (1978). Emotions are expressed more intensely on the left side of the face. *Science, 202*(4366), 434–436. 10.1126/science.705335 705335 10.1126/science.705335

[CR98] Semyonov, O., Ziv-El, A., Krumhuber, E. G., Karasik, S., & Aviezer, H. (2021). Beyond shared signals: The role of downward gaze in the stereotypical representation of sad facial expressions. *Emotion, 21*(2), 247–259. 10.1037/emo0000706 31886681 10.1037/emo0000706

[CR99] Sim, V. L., Guberman, A., & Hogan, M. J. (2005). Acute bilateral opercular strokes causing loss of emotional facial movements. *Canadian Journal of Neurological Sciences, 32*(1), 119–121. 10.1017/S0317167100017005 10.1017/s0317167100017005 15825559

[CR100] Srinivasan, R., & Martinez, A. M. (2018). Cross-cultural and cultural-specific production and perception of facial expressions of emotion in the wild. *IEEE Transactions on Affective Computing,**12*(3), 707–721. 10.1109/TAFFC.2018.2887267

[CR101] Steward, B. A., Mewton, P., Palermo, R., & Dawel, A. (2024). Interactions between faces and visual context in emotion perception: A meta-analysis. *Pyschonomic Bulletin & Review*. 10.3758/s13423-025-02678-6 10.3758/s13423-025-02678-6 PMC12426097 40180758

[CR102] Sutherland, C. A. M., Oldmeadow, J. A., Santos, I. M., Towler, J., Burt, D. M., & Young, A. W. (2013). Social inferences from faces: Ambient images generate a three-dimensional model. *Cognition, 127*(1), 105–118. 10.1016/j.cognition.2012.12.001 23376296 10.1016/j.cognition.2012.12.001

[CR103] Talhelm, T., Oishi, S., & Zhang, X. (2019). Who smiles while alone? Rates of smiling lower in China than U.S. *Emotion, 19*(4), 741–745. 10.1037/emo0000459 29963886 10.1037/emo0000459

[CR104] Talipski, L., Palermo, R., Sutherland, C., Gignac, G., Jeffery, L., Crookes, K., Wilmer, J. B., Krumhuber, E., Bell, J., & Dawel, A. (2025). *Introducing the Naturalistic Expression Recognition Task (NERT): Associations with posed expression recognition, empathy, and general cognitive ability*. 10.31234/osf.io/2xg8z_v1 10.3758/s13428-026-02944-y PMC13076514 41975116

[CR105] Thibault, P., Levesque, M., Gosselin, P., & Hess, U. (2012). The Duchenne marker is not a universal signal of smile authenticity – But it can be learned! *Social Psychology, 43*(4), 215–221. 10.1027/1864-9335/a000122

[CR106] Thorstenson, C. A., Pazda, A. D., & Lichtenfeld, S. (2020). Facial blushing influences perceived embarrassment and related social functional evaluations. *Cognition and Emotion, 34*(3), 413–426. 10.1080/02699931.2019.1634004 31230523 10.1080/02699931.2019.1634004

[CR107] Tottenham, N., Tanaka, J. W., Leon, A. C., McCarry, T., Nurse, M., Hare, T. A., Marcus, D. J., Westerlund, A., Casey, B. J., & Nelson, C. (2009). The NimStim set of facial expressions: Judgments from untrained research participants. *Psychiatry Research, 168*(3), 242–249. 10.1016/j.psychres.2008.05.006 19564050 10.1016/j.psychres.2008.05.006 PMC3474329

[CR108] Trevisan, D. A., Hoskyn, M., & Birmingham, E. (2018). Facial expression production in autism: A meta-analysis. *Autism Research, 11*(12), 1586–1601. 10.1002/aur.2037 30393953 10.1002/aur.2037

[CR109] Uono, S., & Hietanen, J. K. (2015). Eye contact perception in the west and east: A cross-cultural study. *PLoS One, 10*(2), e0118094. 10.1371/journal.pone.0118094 25714900 10.1371/journal.pone.0118094 PMC4340785

[CR110] Urban, P. P., Wicht, S., Marx, J., Mitrovic, S., Fitzek, C., & Hopf, H. C. (1998). Isolated voluntary facial paresis due to pontine ischemia. *Neurology, 50*(6), 1859–1862. 10.1212/WNL.50.6.1859 9633742 10.1212/wnl.50.6.1859

[CR111] Valente, D., Theurel, A., & Gentaz, E. (2018). The role of visual experience in the production of emotional facial expressions by blind people: A review. *Psychonomic Bulletin & Review, 25*(2), 483–497. 10.3758/s13423-017-1338-0 28646269 10.3758/s13423-017-1338-0 PMC5902524

[CR112] Verhoef, T., & Fosch-Villaronga, E. (2023). *Towards affective computing that works for everyone. In 2023 11th International Conference on Affective Computing and Intelligent Interaction (ACII)* (pp. 1–8). 10.1109/ACII59096.2023.10388169

[CR113] Witkower, Z., Rule, N. O., & Tracy, J. L. (2023). Emotions do reliably co-occur with predicted facial signals: Comment on Durán and Fernández-Dols (2021). *Emotion, 23*(3), 903–907. 10.1037/emo0001162 37079837 10.1037/emo0001162

[CR114] Zhang, Z., & Winkielman, P. (n.d.). How do faces move us? Expressions can influence us by changing subjective experience but also via a variety of non-experiential mechanisms. *Affective Science* Special Issue on Face Emotion.

[CR115] Zloteanu, M., & Krumhuber, E. G. (2021). Expression authenticity: The role of genuine and deliberate displays in emotion perception. *Frontiers in Psychology, 11*, 611248. 10.3389/fpsyg.2020.611248 33519624 10.3389/fpsyg.2020.611248 PMC7840656

